# Serial viral load analysis by DDPCR to evaluate FNC efficacy and safety in the treatment of mild cases of COVID-19

**DOI:** 10.3389/fmed.2023.1143485

**Published:** 2023-03-14

**Authors:** Renato Martins da Silva, Paula Gebe Abreu Cabral, Sávio Bastos de Souza, Raul Ferraz Arruda, Sheila Passos de Figueiredo Cabral, Arícia Leone Evangelista Monteiro de Assis, Yolanda Porto Muniz Martins, Carlos Augusto de Araújo Tavares, Antônio Brazil Viana Junior, Junbiao Chang, Pingsheng Lei

**Affiliations:** ^1^High Complexity Center, Galzu Institute, Campos dos Goytacazes, Rio de Janeiro, Brazil; ^2^Universidade Federal do Ceará (UFC)/Ebserh University Hospital Complex, Fortaleza, Brazil; ^3^NMPA Key Laboratory for Research and Evaluation of Innovative Drug, Henan Normal University, Xinxiang, China; ^4^Institute of Material Medical, Chinese Academy of Medical Sciences & Peking Union Medical College, Beijing, China

**Keywords:** COVID-19, SARS-CoV-2, AZVUDINE, FNC, antiviral

## Abstract

**Introduction:**

The SARS-CoV-2 outbreak has threatened the human population globally as the numbers of reinfection cases even after large-scale vaccination. Trials have been carried out to find drugs effective in fighting the disease, as COVID-19 is being considered a treatable disease only after we have antivirals. A clinical candidate originally developed for HIV treatment, AZVUDINE (FNC), is a promising drug in the treatment of COVID-19.

**Methods:**

To predict the clinical outcome of COVID-19, we examined the course of viral load, every 48 h, by RT-PCR, and disease severity using an antiviral drug, FNC, with 281 participants. A randomized clinical trial was performed to evaluate the efficacy of FNC added to standard treatment, compared with placebo group added to standard treatment, for patients with mild COVID-19. RT-qPCR and ddPCR were applied to estimate the viral load in samples from patients. Also, the clinical improvement was evaluated as well as the liver and kidney function.

**Results and discussion:**

Notably, the FNC treatment in the mild COVID-19 patients may shorten the time of the nucleic acid negative conversion (NANC) versus placebo group. In addition, the FNC was effective in reducing the viral load of these participants. The present clinical trial results showed that the FNC accelerate the elimination of the virus in and could reduce treatment time of mild patients and save a lot of medical resources, making it a strong candidate for the outpatient and home treatment of COVID-19.

**Clinical trial registration:**

https://clinicaltrials.gov/ct2/show/NCT05033145, identifier NCT05033145.

## 1. Introduction

A new coronavirus disease-2019 (COVID-19) caused by severe acute respiratory syndrome coronavirus type 2 (SARS-CoV-2) occurred as a pandemic resulting in serious disease burden in almost all countries (1, 2). After 3 years since the first cases of coronavirus disease 2019 (COVID-19) were reported the people have been striving to turn COVID-19 into a preventable and treatable disease through various measures, including vaccines (3, 4), small-molecule inhibitors (5, 6), bioactive natural products (7–9), and traditional medicine (10, 11). The triumph of vaccines has led to a significant decrease in symptomatic illness, severe and critical disease, and death. Nonetheless, the efficacy of vaccines has been affected by virus evolution and the emergence of new variants, and global access is sub-optimal (2).

The development of small molecules to treat COVID-19 has been achieved by several strategies, including computer-aided lead compound design and screening, natural product discovery, drug repurposing, and combination therapy. Antiviral drugs offer opportunities at various stages of SARS- CoV-2 infection, including pre- or post-exposure prophylaxis, early treatment, and late treatment. However, current small molecule antivirals have major limitations, such as remdesivir, in which the efficacy has varied among reports (12–15), the Paxlovid which is expensive, US $530 for each 5-day course (16), and has multiple drug-drug interactions, and the molnupiravir, costing US $700 per 5-day course (16), and has mutagenic potential (17).

Recently published studies illustrated the efficacy and safety of early use of a small-molecule antiviral in reducing hospitalization or death among the high-risk population with mild to moderate COVID-19 (12, 18–20). This promising antiviral, AZVUDINE (FNC), is the first double-target nucleoside drug that has demonstrated significant and broad-spectrum *in vitro* antiviral effects against targets such as HIV (21), HCV (22), EV71 (23), HBV (24), and recently a randomized, open-Label, controlled clinical trial of FNC tablets was performed in the treatment of mild and common COVID-19, showing that FNC treatment in the mild and common COVID-19 may shorten the mean times of the first nucleic acid negative conversion (NANC) versus standard antiviral treatment (25).

To detect and diagnose the virus, reverse transcription quantitative real-time polymerase chain reaction (RT-qPCR) is applied in many countries, and this method can estimate the viral load in samples from patients with this viral infection (13). Recently, Tsukagoshi et al. (18) examined viral loads of SARS-CoV-2 in fatal (15 cases), symptomatic/survived (133 cases), and asymptomatic cases (138 cases) using RT-qPCR. Notably, the viral load in the fatal cases was significantly higher than in symptomatic or asymptomatic cases (*p* < 0.05). The authors conclude that intervene early to prevent a severe stage of the disease in such cases.

Therefore, clinical trials of FNC treating COVID-19 with larger sample size were required. Thus, the objective of this randomized clinical trial was to evaluate the efficacy of FNC, evaluating the clinical improvement, the liver and kidney function, the NANC time, and the viral load in mild COVID-19 participants.

## 2. Methodology

### 2.1. Study design

This randomized, double-blind, placebo-controlled clinical trial (IGZ-2) was carried out in five co-participating research centers, distributed in four municipalities in the state of Rio de Janeiro, Brazil, as a strategic decision due to the need to concentrate molecular biology analyzes to maintain their standardization and quality, remembering that each RT-PCR equipment has its own sensitivity and different kits of reagents for RT-PCR have different performances. Two research centers were located in the city of Campos dos Goytacazes, namely Hospital São José and Santa Casa de Miseriocordia de Campos. In the municipality of São fidelis, the third research center was at Hospital Armando Vidal. In the municipality of Itaocara, the fourth research center was located at the Hospital de Itaocara. And the fifth research center was located in the municipality of Cambuci at Hospital Moacyr Gomes de Azevedo. However, the research centers served as an outpatient clinic for the evaluation of the participants, since this study treated mild patients contaminated with SARS-COV-2, all of whom were followed in their homes. This study took place in January 2022 when the Centers for Combating COVID-19 (CCC) provided the first care for infected patients. Interestingly, in this study, 88% of participants were vaccinated and 12% had natural immunity. Participants from four co-participating centers in the north and northwest regions of Rio de Janeiro were randomized. This trial was approved by the institutional review board of the National Health Surveillance Agency CE 0937457/21-4. The study was approved by the National Council for Research Ethics, CAAE 52176421.8.0000.5244. The study was also published in clinical trials (NCT05033145). All enrolled participants provided written informed consent. The methods are described in detail in the Supplementary material.

Patients in the FNC group were treated with oral AZVUDINE tablets 5 mg (five tablets once a night) and standard treatment. For the 5 mg dose of AZVUDINE, the mean half-life is 13.8 h, with the intact drug and metabolites being excreted in the urine within 24 h. Patients in the control group were treated with placebo added standard treatment.

Patients: Patients meeting the following criteria were enrolled in the study: (1) age 18 and over, regardless of gender; (2) respiratory or blood samples that were tested positive for SARS-CoV-2 nucleic acid by RT-PCR, or respiratory or blood samples that were tested highly homologous with the known SARS-CoV-2 by viral gene sequencing; (3) the confirmation of COVID-19 according to the diagnostic criteria of “the latest Clinical guide-lines for novel coronavirus” issued by the World Health Organization (WHO) on 28 January 2020. All enrolled patients signed informed consent forms.

Exclusion criteria included (1) known or suspected allergy to the com- position of AZVUDINE tablets; (2) patients with malabsorption syndrome or any other condition that affects gastrointestinal absorption, the need for intravenous nutrition or an inability to take oral medication; (3) patients on anti-HIV treatment; (4) patients with one of the following conditions: Respiratory failure and the need for mechanical ventilation; shock; intensive care unit (ICU) monitoring and treatment for other organ failures; (5) pregnant women or those who were lactating or may have a birth plan during the trial period and within 6 months after the end of the trial; (6) patients participating in other clinical trials or using experimental drugs within 12 weeks before administration; and (7) patients with other conditions that were not suitable for participating in this experiment according to the judgment of the researcher.

The definition of mild COVID-19 the definition of common COVID-19 was patients with fever, respiratory and characteristic symptoms such as loss of smell, loss of taste, diarrhea, dizziness, fever, chill, sore throat, dyspnea, tachypnea, nausea, and abdominal pain.

Enrollment: Volunteers were approached at health posts and testing points for SARS-COV-2. Patients with fever, cough or other symptoms related to COVID-19 were approached by the study team, who provided an explanation of all pertinent information. After this process the ICF was applied and samples were collected for the nucleic acid test by RT-PCR for laboratory tests. The following day, the researcher evaluated the results of the exams and verified whether the volunteer met the inclusion criteria, thus proceeding to the randomization stage.

Randomization: After the patients signed the consent form, exams were performed to check whether they would attend the eligibility criteria, and this assessment was performed by the main investigator. If the patients were accepted as participants of the study, the research coordinator randomized the participants into the system (Researcher IGZ v2.0 Software). After being randomized, the participants received the randomization numbers, entering one of the study groups (blind). Thus, this information went to different sectors such as pharmacy and infirmary. Patients were randomly assigned in a 1:1 ratio to the FNC group or control group. Randomization was accomplished by using a random table that was generated in Researcher IGZ v2.0 Software at 1:1. Each enrolled subject was given a number, randomly assigned to the FNC group and control group according to a predetermined random table and received treatment according to the corresponding treatment regimen.

Procedures: Patients in the FNC group were treated with oral AZVUDINE tablets 5 mg d^–1^ (five tablets once a night) and symptomatic treatment. The FNC dose was determined due COVID-19 clinical trials preliminary results, considering that the maximum safety dose study of AZVUDINE was performed for 5 mg, a daily dose of 5 mg may meet clinical treatment. In addition, for the 5 mg dose of AZVUDINE, the mean half-life is 13.8 h, with the intact drug and metabolites being excreted in the urine within 24 h. Patients in the control group were treated with placebo added standard treatment.

The patient’s vital signs, oxygen (*via* finger pulse oximetry), and respiratory symptoms and signs were monitored every day. On odd days and the discharge day, the patient’s routine blood, erythrocyte sedimentation rate (ESR), C-reactive protein, blood biochemistry, blood coagulation, myocardial markers, procalcitonin, myocardial zymogram, and arterial blood gas were monitored. SARS-CoV-2 nucleic acids were tested by RT-PCR after the patients began taking their drugs. Nucleic acid detection analyzes were performed every 48 h days throughout the treatment period for optimal measurement of participant’s viral load. Two consecutive negative results configured clinical discharge. These tests were used to obtain the mean times of the nucleic acid negative conversion (NANC).

Primary outcome: The primary outcome was the proportion of patients hospitalized during the study through day 28, according to the WHO Ordinary Clinical Progression Scale (Jun/2020), Score 4–10.

Secondary outcomes: Proportion of participants with a clinical outcome of CURE during the study; Improvement in clinical status in at least one category compared to screening on the Ordinal Scale of Clinical Improvement (WHO, Jun/2020); Severity and duration of symptoms: fever, cough, fatigue or tiredness, breathlessness, myalgia, nasal congestion or runny nose, sore throat, headache, chills, nausea, vomiting, anosmia, ageusia; Baseline changes in liver and kidney function; Time of use of AZVUDINE until the second negative conversion of RT-PCR; Evaluation of SARS-CoV-2 viral load negative conversion time by RT-PCR between AZVUDINE group (FNC) and control group; Evaluation of the number of cycles for the detection of SARS-CoV-2 viral load by RT-PCR and application of the standard curve to calculate the viral load; Analysis of the relationship between the calculated and/or quantified viral load and the clinical evolution of the participants in the AZVUDINE group (FNC) in relation to the control group; Frequency and intensity of adverse events, unexpected adverse events, and serious adverse events.

Safety was regularly assessed by monitoring vital signs, changes in laboratory values (liver function and renal function), and adverse events (including type, incidence, severity, time and drug correlation, and assessment of severity). Previous studies have already shown that individuals who used FNC did not experience any type of serious adverse event drug related (25).

### 2.2. Quantification of SARS-COV-2 viral load by reverse transcription–polymerase chain reaction (RT-PCR)

Total RNA was extracted using the MagMAX™ Viral/Pathogen Nucleic Acid Isolation kit (Applied Biosystems) according to the manufacturer’s instructions, using nasal and throat swabs from the clinical study participants. Once total RNA was extracted, RT-PCRs were performed using the TaqPath™ COVID-19 CE-IVD RT-PCR kit (ANVISA Reg: 10358940107) according to the manufacturer’s instructions, using the QuantStudio5 RT-PCR equipment, Applied Biosystems, (ANVISA Reg: 10358940069). The primers and probes targeted the ORF1ab and N genes.

A standard curve was constructed using serial dilutions of the positive control (TaqPath COVID-19 Control), which is SARS-CoV-2 viral RNA at a known concentration of 1 × 10^4^ copies/μL. The CTs obtained from each sample by RT-PCR were plotted on the standard curve to estimate the viral load of each sample.

A positive RT-PCR result occurs in CTs ≤ 37. In this case, when viral RNA is present, the specific probe used to detect SARS-CoV-2 is broken by DNA polymerase, emitting fluorescence. High copy number of viral RNA generates high levels of fluorescence, therefore, the CT value appears earlier during the reaction. Low copy number of viral RNA generates low level of fluorescence, and consequently, the CT value appears later. Values of CTs > 37 are considered negative. By establishing a viral RNA concentration curve (present in the positive control), we will obtain a curve of CTs, from lower values (higher copies of viral RNA) to higher values (lower copies of viral RNA).

### 2.3. Quantification of SARS-COV-2 viral load by droplet digital polymerase chain reaction (DDPCR)

Total RNA was extracted using the MagMAX™ Viral/Pathogen Nucleic Acid Isolation kit (Applied Biosystems) according to the manufacturer’s instructions, using nasal and throat swabs from the clinical study participants. Once total RNA was extracted, the ddPCR were performed subsequently.

Reverse transcription–PCR was conducted with primers and probes targeting the ORF1ab and N genes and a positive reference gene. Reaction system and amplification conditions were performed according to the manufacturer’s specifications (Shanghai BioGerm Medical Technology Co., Ltd., China). The result was considered valid only when the cycle threshold (Ct) value of the reference gene was 38 or less. The result was considered positive when the Ct values of both target genes were 38 or less and negative when they were both greater than 38. If only one of the target genes had a Ct value of 38 or less and the other was more than 38, it was interpreted as a single-gene positive.

Digital droplet PCR analyzes were performed by the Targeting One Digital PCR System; COVID-19 digital PCR detection kit; droplet generation Kit; Droplet detection kit. The kits allow detection of the ORF1ab gene, N gene and a positive reference gene. The detection limit was 10 copies/test (Targeting OneTechnology is licensed by the China Food and Drug Administration).

### 2.4. Statistical analysis

For the analysis of demographic information and baseline eigenvalues, the mean value, standard deviation, quartiles, minimum, and maximum values for numerical variables were calculated. For categorical data, frequency and percentage were calculated. The comparison of the two groups under general conditions was analyzed with appropriate methods according to the types of indicators. The Mann–Whitney test was used to compare the groups regarding quantitative data. Fisher’s exact test was used for categorical data. Statistical analyzes were performed using the R-studio software.

## 3. Results and discussion

### 3.1. Demographic analysis

**FIGURE 1:**
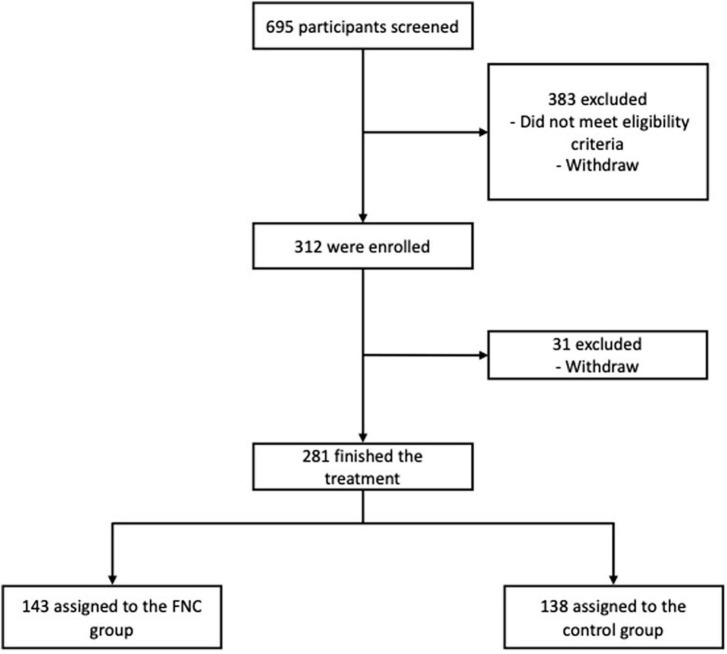
Trial profile.

A total of 695 participants were selected for this study between the months of January and May of the year 2022. Of these, 383 were excluded for not meeting the eligibility criteria or for dropping out before participating in the clinical trial. A total of 312 participants were randomized, of which 281 completed the treatment successfully and 31 dropped out before completing treatment (Figure 1).

Patient demographics and baseline characteristics were well matched between the FNC group and the control group at enrollment (Supplementary Table 1). The median age was 44 years (IQR 32–56), with the median age for men being 43 years (IQR 31–58) and for women 46 years (IQR 34–54). The largest number of participants was female, totaling 170 individuals (60.5%) (Supplementary Table 1).

### 3.2. Clinical improvement

The results indicates that absolutely all study participants started with a score three (Supplementary Table 2 and Supplementary Figure 1). At the time of clinical discharge, except for the withdrawal patients, all ended up with a score of 0 or 1, according to the WHO Clinical Improvement Ordinal Scale. Although the shorter sample size clinical trial of FNC did not use the WHO Clinical Improvement Ordinal Scale, all participants of the study were discharged without viral RNA detection, or they were asymptomatic with viral RNA detected, which would be equivalent to scores 0 and 1, respectively (14). Both FNC and placebo patients showed statistically significant differences between baseline and final status (Supplementary Table 2). However, it was not possible to observe the significant difference between the groups in relation to the initial score (*p* = 0.999) and final score (*p* = 0.700) (Table 1 and Supplementary Table 2).

**TABLE 1:** Compilation of results of contrasts between FNC and placebo treatments on study outcomes.

Objective and outcomes	*P*-value
Score reduction	>0.999
Cure time/absence of viral RNA	<0.001
Viral load negative time	<0.001
Days 1st negative conversion	<0.001
Days 2nd negative conversion	<0.001
Medication use time	<0.001
Moment of viral load reduction	D3, D5, D9, D11
Participants who did not become viral negative within 14 days	1.77%

The results showed that the proportion of FNC participants who presented Score 1 at clinical discharge was 2.0%, with 98% presenting a score 0. In relation to placebo, 2.2% of the participants presented a score 1 in clinical discharge, and 97.8% presented Score 0 (Supplementary Table 2 and Table 1). It is reported that COVID-19 is basically a self-limiting viral infection, and it resolves gradually over time, especially for mild cases (17), so it is not surprising that no statistical difference is observed between the scores of the groups.

### 3.3. Time of the nucleic acid negative conversion

**FIGURE 2:**
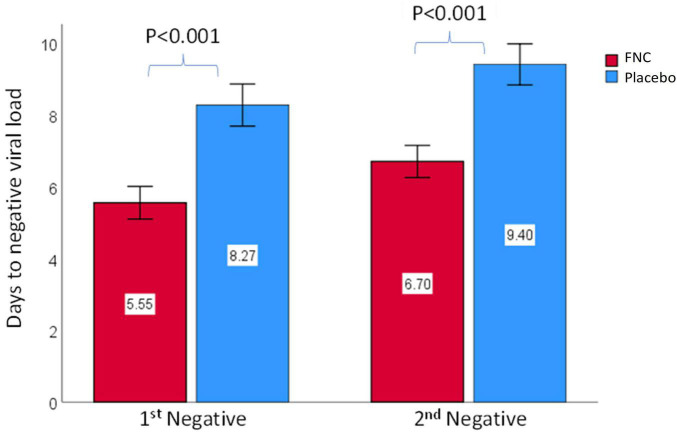
Comparison of the time of the first and the second nucleic acid testing negativity between all subjects in the FNC group and the placebo group. Data are mean (SD). The differences between groups were analyzed using Mann–Whitney test.

The mean times of the nucleic acid negative conversion (NANC) could reflect the efficacy of drugs and was used as a marker of clinical improvement in this study, in which two consecutive negative results configured clinical discharge. The results indicate that participants treated with FNC had a significantly shorter time to first nucleic acid negative conversion (5.55 days; *p* < 0.001) compared to participants treated with placebo (8.27 days) (Figure 2). The same was repeated in the results obtained for the second nucleic acid negative conversion, in which the individuals treated with FNC had a shorter time for the consecutive negative (6.7 days; *p* < 0.001) compared to participants treated with placebo (9.40 days) (Figure 2). Although there were no significant differences regarding the final score between the groups (Table 2 and Supplementary Figure 1), the time taken for FNC individuals to be discharged from the clinic was significantly shorter, therefore, despite practically all participants having completed treatment with a score 0, regardless of the drug used, it is noted that the participants who were treated with FNC had a shorter time to cure and, consequently, were discharged from the clinic (Figure 2). These data reinforce what was observed by Ren et al. (25), which showed that FNC treatment in the mild and common COVID-19 may shorten the NANC time versus standard antiviral treatment. Therefore, FNC could reduce treatment time of mild patients and save a lot of medical resources.

**TABLE 2:** Absolute viral load analysis by RT-PCR and DDPCR during the treatment days.

Variables	*N*	Total^1^	Azvudine (*N* = 143^1^)	Placebo (*N* = 138^1^)	*P*-value^2^
Viral load (D0)	281	9.971 ± 4.257 (11.553)	10.239 ± 3.985 (11.593)	9.694 ± 4.519 (11.394)	0.543
Viral load (D1)	275	8.631 ± 4.897 (10.756)	8.359 ± 5.083 (10.834)	8.909 ± 4.702 (10.717)	0.634
Viral load (D3)	275	5.733 ± 5.503 (1.037)	4.452 ± 5.564 (969)	7.023 ± 5.146 (10.000)	<0.001
Viral load (D5)	242	4.285 ± 5.189 (971)	3.016 ± 4.897 (0)	5.513 ± 5.187 (9.642)	<0.001
Viral load (D7)	186	1.773 ± 3.613 (63)	1.483 ± 3.516 (0)	1.987 ± 3.684 (967)	<0.001
Viral load (D9)	119	1.276 ± 3.282 (0)	1.439 ± 3.653 (0)	1.214 ± 3.149 (29)	0.064
Viral load (D11)	70	556 ± 2.230 (0)	902 ± 2.637 (0)	462 ± 2.124 (0)	0.712
Viral load (D13)	41	334 ± 1.526 (0)	501 ± 536 (491)	294 ± 1.685 (0)	0.002
DDPCR (D1)	279	36.473 ± 69.608 (7.004)	31.452 ± 64.185 (5,672)	41.751 ± 74,764 (11.619)	0.220
DDPCR (D3)	268	25.715 ± 57,361 (3,114)	13,063 ± 36,706 (638)	38.749 ± 70.572 (11.466)	<0.001
DDPCR (D5)	219	23.461 ± 56,423 (1,141)	3,828 ± 15,666 (0)	38,783 ± 70,409 (9,441)	<0.001
DDPCR (D7)	165	12,468 ± 34,138 (107)	1,454 ± 5,569 (0)	18,761 ± 41,355 (2,456)	<0.001
DDPCR (D9)	110	11,831 ± 39,217 (77)	10,298 ± 52,889 (0)	12,433 ± 32,742 (880)	<0.001
DDPCR (D11)	60	3,645 ± 11,721 (26)	379 ± 686 (0)	4,378 ± 12,875 (72)	0.153
DDPCR (D13)	36	329 ± 1,525 (0)	394 ± 735 (0)	313 ± 1,670 (0)	0.049

^1^Mean ± standard deviation (Median).

^2^Mann–Whitney test; Wilcoxon rank sum exact test.

### 3.4. Detection of SARS-COV-2 viral load by RT-PCR and DDPCR technique

**FIGURE 3:**
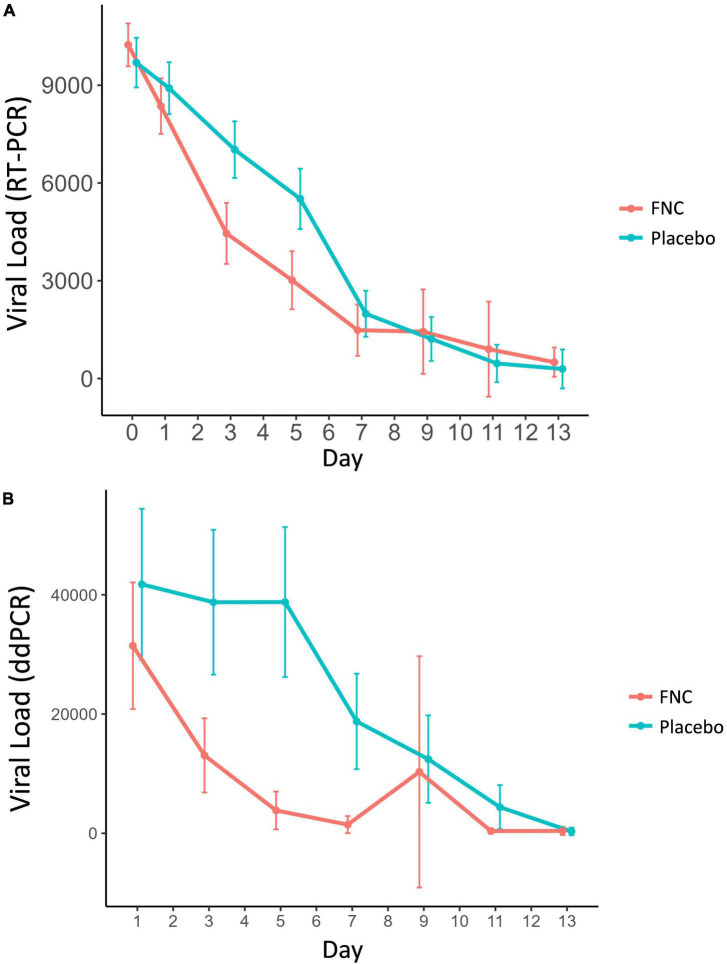
Viral load analysis measured by RT-PCR **(A)** and DDPCR **(B)** of all participants in the FNC group and the placebo group. Data are median (SD) (Red line: FNC; Blue line: PLACEBO).

In this study the FNC group showed a marked increase in CTs/day when compared to the placebo group (Supplementary Table 3 and Supplementary Figure 2). It was possible to observe significant differences between the two groups mainly on days D3, D5, and D7 (*p* < 0.001). After D7, the viral loads (measured by RT-PCR) found in the participants of the two groups tend to be equal (Supplementary Table 3 and Figure 3A). This result is an important fact, since it demonstrates that the FNC was effective in reducing the viral load of these participants, especially in the first days (Supplementary Table 3 and Figure 3A). Other antivirals do not have the same effectiveness in reducing viral load as FNC.

Goldberg et al. (14) and Wang et al. (13) demonstrated that remdesivir treatment of COVID-19 patients did not significantly reduce nasopharyngeal viral load. Other antiviral, molnupiravir, may not reduce the virus replication effectively after the first 24 h of COVID-19 infection (17), and the administration of high doses of favipiravir (300 and 500 mg/kg) showed a reduction in virus load. In addition, the combination therapy of molnupiravir and favipiravir increases the number of mutations in the RNA structure dramatically compared with favipiravir or molnupiravir alone, which in turn significantly reduces the RNA titer (17).

In the case of Paxlovid, the viral load is reduced after 5 days compared with placebo (26). Although patients could benefit from Paxlovid, they may be at significant risk for drug interactions and harm owing to the ritonavir component of Paxlovid, a particularly potent inhibitor of cytochrome P450 system CYP3A enzymes, leading to dangerous interactions between ritonavir and CYP3A-dependent drugs (27). Therefore, it is a consensus that the Paxlovid has a high risk of drug-drug interaction (27–29). Therefore, FNC treatment can not only accelerate the elimination of the virus but also reduce the time of infection since the time of nucleic acid negative conversion is shortened.

Similar to the viral load results obtained by the RT-PCR technique, however, with greater intensity, a great difference between the two groups was observed in the viral load quantified by the DDPCR technique (Table 2 and Figure 3B), in which it is possible to observe that participants who ingested FNC had a marked decline in SARS-CoV-2 viral load/day when compared to participants who took placebo (Table 2 and Figure 3B). In Table 2, we observe that already on the 3rd day of treatment there is a large statistical difference between the participants who ingested FNC and placebo (*p* < 0.001), which becomes even greater on the 5th day of treatment (*p* < 0.001).

The fact that the investigational product promotes a rapid drop in the viral load of the participants is a strong indicator that it has a greater probability of clinical improvement (30, 31) reducing the chances of aggravation of the case by the progression of the disease.

### 3.5. Participants vaccinated for COVID-19 during the study

**FIGURE 4:**
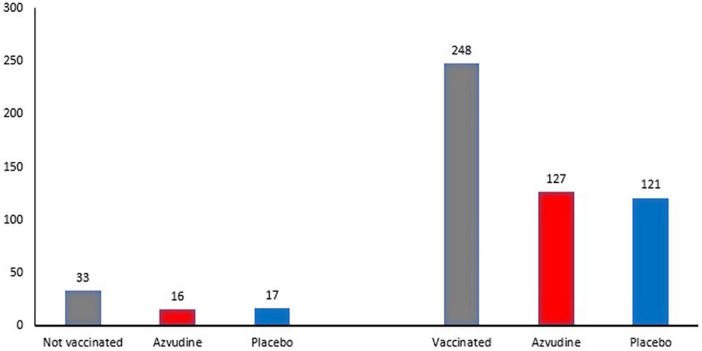
Quantification of vaccinated and unvaccinated participants included in the study and its distribution between the FNC and placebo groups.

Vaccines have been used to contain the spread of COVID-19, however, the numbers of reinfection cases increase even after large-scale vaccination (32). Some studies summarize the problems associated vaccines development for COVID-19 and conclude by a need to study deeply on the structure, mutations, and function of COVID-19 as well as its pathophysiology from a large population (3, 4). This clinical trial has 88% of participants vaccinated and reinfected by COVID-19. Only 12% had natural immunity (Table 3 and Figure 4). Still, there was efficacy of FNC independent of vaccination.

**TABLE 3:** Quantification of vaccinated and unvaccinated participants included in the study.

Variables	*N*	Total^1^	Azvudine (*N* = 143^1^)	Placebo (*N* = 138^1^)	*P*-value^2^
Participants	281				0.769
Vaccinated	248	248 (88%)	127 (89%)	121 (88%)	
Not vaccinated	33	33 (12%)	16 (11%)	17 (12%)	

^1^*n* (%); Median (IQR).

^2^Pearson’s chi-square test.

### 3.6. Time to improvement of symptoms

The time of improvement of the symptoms evaluated during the study was measured through the number of days in which the participants remained presenting the symptom. Symptoms characteristic of patients infected with the SARS-CoV-2 virus were evaluated (Supplementary Table 4). Here, there is no statistical difference in the symptom’s characteristic between the two groups, FNC and placebo, of participants. These results corroborate the previously published FNC pilot study treating COVID-19, where Ren et al. (25) also demonstrated no difference in symptoms and laboratory test results during the screening between the FNC group and the control group.

### 3.7. Changes in kidney and liver functions baselines

**FIGURE 5:**
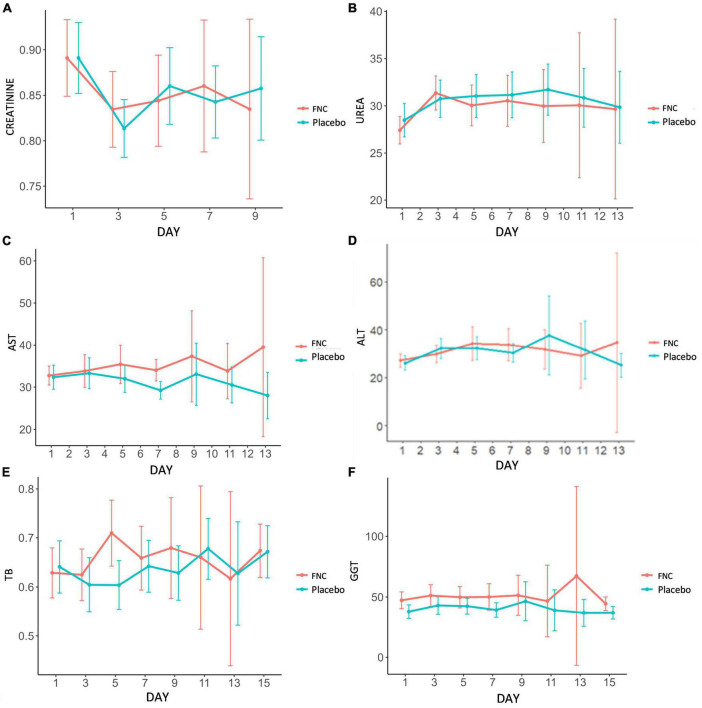
During the treatment, the dynamic changes in kidney and liver markers: **(A)** Creatinine, **(B)** urea, **(C)** ALT, **(D)** AST, **(E)** BT, and **(F)** GGT of the patients in the FNC group and patients in the placebo group. Data are median (SD) (Red line: FNC; Blue line: PLACEBO).

It is much discussed about kidney and liver damage associated with administration of antivirals in ongoing clinical trials for COVID-19 (15, 33, 34). For the COVID-19 patients treated with others antivirals as remdesivir, poor renal and liver function were both exclusion criteria in randomized clinical trials and contraindication for treatment (35–37). In the case of tenofovir, nephrotoxicity and hepatoxicity were also reported as adverse drug events occurred after long-term treatment. In this study, FNC treatment was well tolerated for patients. In long-term studies (48 weeks), FNC was shown to be safe in the treatment of HIV (38). The vital signs, liver function and kidney function in both groups were normal. The results of the tests referring to the renal function of the individuals distributed in FNC and in placebo, including creatinine and blood urea nitrogen, showed profiles of similar values, within the normal parameters throughout the treatment and without significant differences between the groups during the days of treatment (Figures 5A, B).

Regarding the results of the tests referring to the liver function of the individuals distributed in FNC and placebo, including aspartate aminotransferase, alanine aminotransferase, glutamyl transpeptidase and total bilirubin, they presented values within the normal range, with the groups presenting similar results profiles and without significant changes during the days of treatment, as well as the results observed in tests referring to renal function. These results demonstrate that the drug is well tolerated by the liver (Figures 5C–F). Therefore, FNC has been shown to be safe in terms of nephrotoxicity and hepatotoxicity and no adverse events have been reported. Moreover, Ren et al. (25) showed that three secondary adverse events were observed in the control group (not treated with FNC).

### 3.8. Adverse events and clinical safety of FNC

A total of 223 cases of adverse events were observed in this study, of which 222 were considered non-serious adverse events and only one was considered a serious adverse event. This single serious adverse event was due to one of the participants having become pregnant in the follow-up stage (after the treatment period), so she was no longer using the medication (Table 4).

**TABLE 4:** Global quantification of adverse events.

*N* = 312	Case	Subject (%)
Adverse events	222	71.15
Severe adverse event (pregnancy)	1	0.32
Adverse event that caused drop-out	0	0
Data expressed in *n* (%)		

The adverse events observed in this study were mainly related to the occurrence of headache (36 cases), Dizziness (35 cases), AST increase (21 cases), Nausea (19 cases), ALT increase (14 cases), and D Dimer increase (11 cases), with normalization of these events until the end of treatment (Table 5). In Table 5 it is possible to compare the adverse events of the FNC and placebo groups, showing a balance in the amount of each adverse event in the two groups, showing a good safety for FNC. The adverse reactions observed in this study were the same as those related to antiviral drugs, with no unexpected adverse reactions (Table 5). Under these conditions, it was possible to observe an increase and a reduction in GGT, but the values tended to decrease even under normal conditions. In this study, 35 cases of dizziness were recorded. Clinical manifestations of COVID-19 include symptoms of vertigo and dizziness since that SARS-CoV-2 neurotropism may inflict a broad spectrum of neuropathic effects (39). Therefore, this adverse event may be related to the disease itself and not caused by FNC. In addition, dizziness could be related to Labyrinthitis (Past history), hypoglycemia (due to loss of taste), Dyspnea, Diarrhea, Cough, Tachypnea, Arterial Hypertension, Tachycardia, and Asthenia too.

**TABLE 5:** Consolidated report of adverse events.

Comparation	Azvudine (*N* = 156)		EAs = 110		Placebo (*N* = 156)		EAs = 112	
Classification	Grade 1		Grade 2		Grade 1		Grade 2	
	Case	Subject (%)	Case	Subject (%)	Case	Subject (%)	Case	Subject (%)
Headache	17	10.89	0	0	19	12,17	0	0
Dizziness	16	10.25	0	0	19	12.17	0	0
AST increase	11	7.05	0	0	10	6.41	0	0
Nausea	10	6.4	0	0	9	5.76	0	0
Alt increase	8	5.12	0	0	6	3.84	0	0
Dimer increase d	4	2.56	0	0	7	4.48	0	0
Hyperglycemia	4	2.56	0	0	5	3.20	0	0
IB increase	4	2.56	0	0	4	2.56	0	0
GGT increase	5	3,20	0	0	3	1.92	0	0
PCR-US reduction	1		0	0	7	4.48	0	0
GGT reduction	4	2.56	0	0	2	1.28	0	0
AST reduction	2	1.28	0	0	3	1.92	0	0
BD increase	3	1.92	0	0	1	0.64	0	0
BD reduction	1	0.64	0	0	2	1.28	0	0
ALT reduction	2	1.28	0	0	1	0.64	0	0
Potassium increase	3	1.92	0	0	0	0	0	0
Palpitation	0	0	1	0.64	0	0	1	0.64
CPK-MB increase	0	0	0	0	2	1.28	0	0
Chest pain	0	0	0	0	2	1.28	0	0
Pregnancy	0	0	1	0.64	0	0	0	0
Hypotension	1	0.64	0	0	1	0.64	0	0
Leukocytes increase	2	1.28	0	0	0	0	0	0
PCR-US increase	1	0.64	0	0	0	0	0	0
IB reduction	0	0	0	0	1	0.64	0	0
Lymphocyte reduction	1	0,64	0	0	0	0	0	0
Arrhythmia	0	0	1	0.64	0	0	0	0
Precordial pain	0	0	0	0	0	0	2	1.28
Abdominal pain	1	0.64	0	0	0	0	0	0
Backache	0	0	0	0	1	0.64	0	0
Bradicardia	0	0	0	0	0	0	1	0.64
Arterial hypertension	0	0	0	0	1	0.64	0	0
Epigastralgia	0	0	0	0	1	0.64	0	0
Anxiety	1	0.64	0	0	0	0	0	0
Sodium reduction	1	0.64	0	0	0	0	0	0
Potassium reduction	1	0.64	0	0	0	0	0	0
Tremor in hands	1	0.64	0	0	0	0	0	0
Skin forming	0	0	0	0	1	0.64	0	0
CPK-MB Reduction	1	0.64	0	0	0	0	0	0
Troponin I reduction	1	0.64	0	0	0	0	0	0
Total partial	107	67.87	3	1.92	108	69.15	4	2.56

## 4. Conclusion

Nowadays, mild cases are the most proportion of COVID-19, the major source of COVID-19 transmission. The present clinical trial results showed that the FNC treatment of mild COVID-19 patients may shorten the time of nucleic acid negativity conversion versus placebo group. For newly diagnosed patients, the time of consecutive nucleic acid negativity conversion was shortened by an average of 2.7 days after treatment with FNC versus placebo group. Also, the FNC treatment can accelerate the elimination of the virus and consequently reduces the mortality of individuals. Despite the availability of vaccines and antivirals today, the pandemic is not under the control and resurfaces in different waves of infection, the pandemic is not under the control and resurfaces in different waves of infection, which cause a large cumulative expense of medical resources since the available antivirals (Paxlovid, Remdesivir, and Molnupiravir) have a high cost and variable efficiency. Fortunately, FNC could reduce treatment time of mild patients and save a lot of medical resources.

## Data availability statement

The data presented in this study are deposited in the Figshare repository (https://figshare.com), accession numbers: https://doi.org/10.6084/m9.figshare.22178723.v1, https://doi.org/10.6084/m9.figshare.22178744.v1, and https://doi.org/10.6084/m9.figshare.22220275.v1. Further inquiries can be directed to PG, pgacabral99@gmail.com.

## Ethics statement

The studies involving human participants were reviewed and approved by the Comissão Nacional de Ética em Pesquisa (CONEP). The patients/participants provided their written informed consent to participate in this study. Written informed consent was obtained from the individual(s) for the publication of any potentially identifiable images or data included in this article.

## Author contributions

PG coordinated the project and supervised the writing of the manuscript. RS and SS performed the analysis of the data and wrote the manuscript. AV assisted in the acquisition of statistical data. RS, RA, SC, AA, and YM assisted in the acquisition of data. CT assisted the medical team that conducted the clinical research. JC and PL assisted in reviewing the manuscript. All authors read and approved the final version of the manuscript.
