# Supplementary material for: Serial viral load analysis by DDPCR to evaluate FNC efficacy and safety in the treatment of mild cases of COVID-19
Source: Front Med (Lausanne). 2023 Mar 14;10:1143485. doi: 10.3389/fmed.2023.1143485 (PMC10053779; doi:10.3389/fmed.2023.1143485)
Supplement: Supplementary file 1 [file Data_Sheet_1.pdf]

SUPPLEMENTARY INFORMATION

1. SUPPLEMENTARY RESULTS

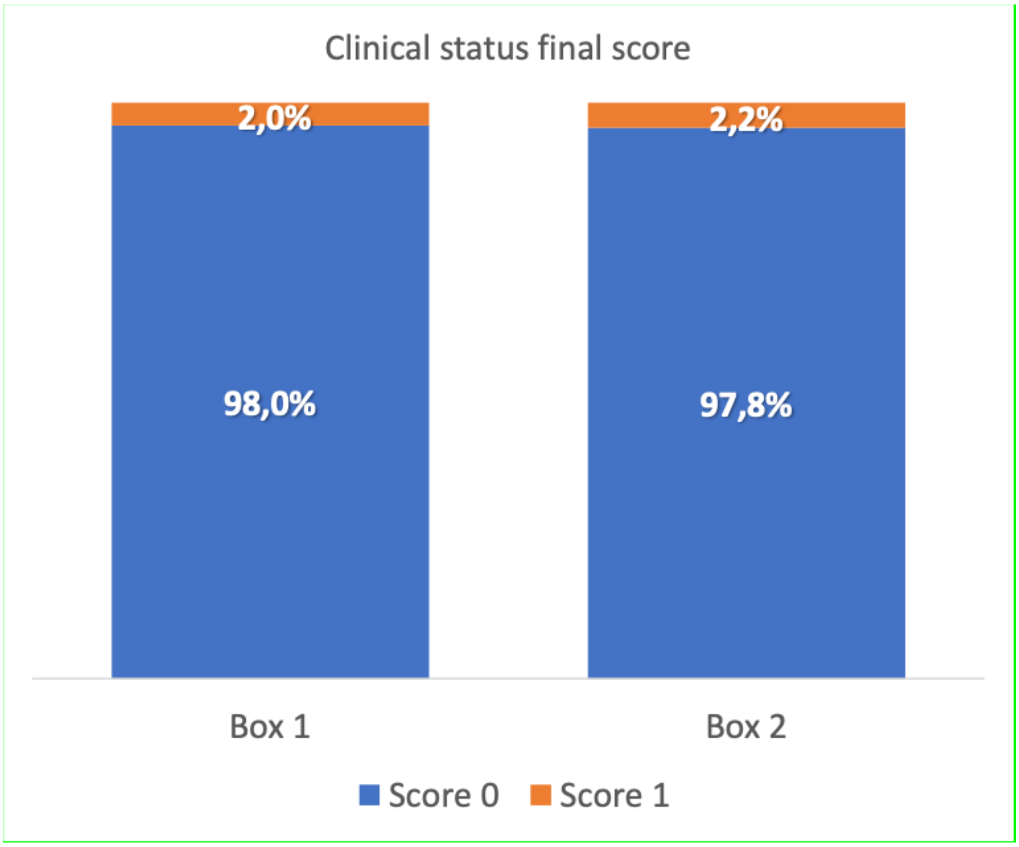

Supplementary Figure 1: Proportion of the initial and final clinical score of all subjects in the FNC group and the placebo group. The p value represents the significant difference between groups by the Wilcoxon test (BOX1: FNC; BOX 2: CONTROL).

CLINICAL CONDITION	DESCRIPTION	SCORE
Non-infected	Without detection of viral RNA	0
Outpatient: mild illness	Asymptomatic; viral RNA detected	1
	Symptomatic; does not need assistance	2
	Symptomatic; needs assistance	3
Hospitalized: moderate illness	Hospitalized; without oxygen therapy	4
	Hospitalized; oxygen support by mask or nasal catheter	5

Supplementary Figure 2: scores of the study related to clinical picture.

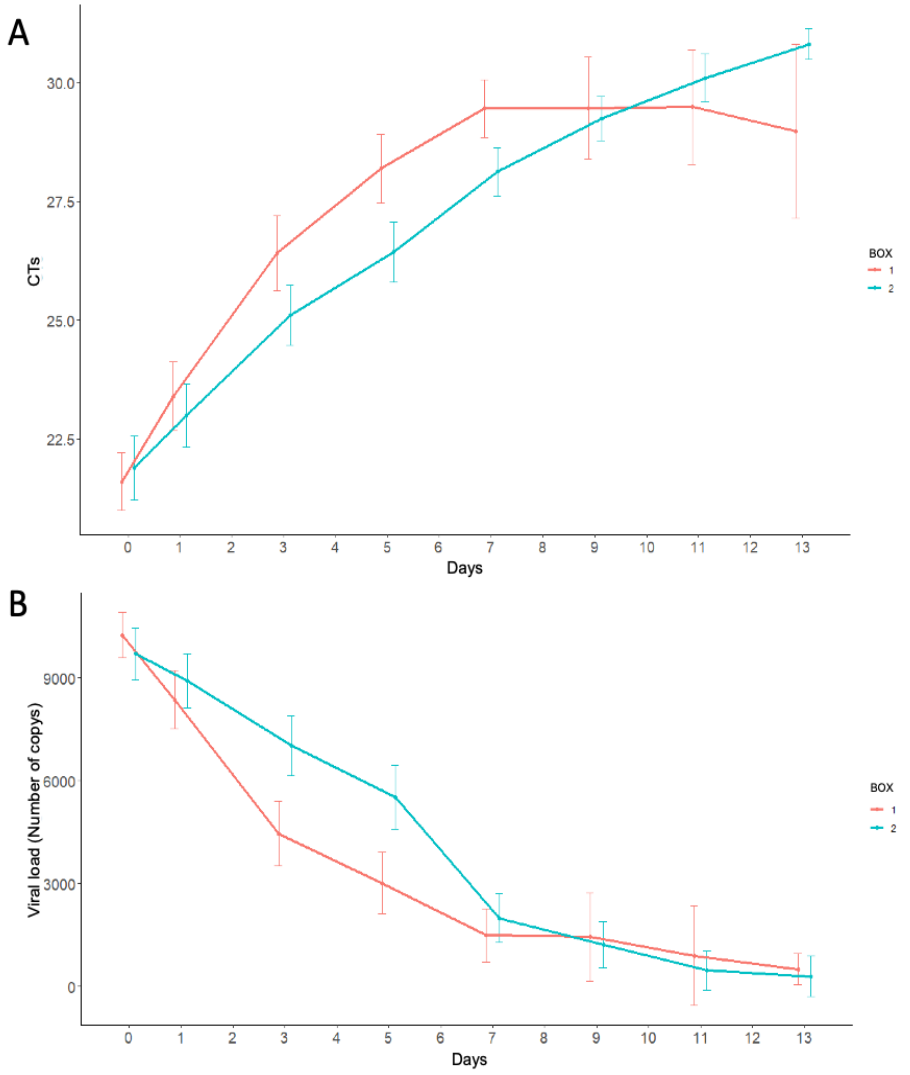

Supplementary Figure 3: Ciral load (B) analysis measured by RT-PCR of all participants in the FNC group and the placebo group. Data are median (SD).

Supplementary Table 1: Demographic and baseline characteristics of participants.

**VARIABLES**	**Total**[1]	AZVUDINE N = 143[1]	PLACEBO N = 138[1]	**p-value**[2]
Age	45 ± 16 (44)	45 ± 16 (43)	45 ± 15 (46)	0.845
Racial classification				0.082
White	169 (60%)	77 (54%)	92 (67%)	
Black	34 (12%)	19 (13%)	15 (11%)	
Brown	78 (28%)	47 (33%)	31 (22%)	
Gender				0.391
F	170 (60%)	83 (58%)	87 (63%)	
M	111 (40%)	60 (42%)	51 (37%)	

[1]*Median (IQR); n (%) /* [2]*Wilcoxon rank sum test; Pearson's Chi-squared test*

Supplementary Table 2: Mean score index (WHO Clinical Improvement Ordinal Scale) of participants at the beginning and final of the clinical research.

	Box 1	Box 2	p^a
Clinical status initial score	3,0	3,0	0,999
Clinical status Final score	0,02 ± 0,14 (0)	0,01 ± 0,12 (0)	0,700
p^b	**<0,001**	**<0,001**	

a:Mann-Whitney U test; b: Wilcoxon test

Supplementary Table 3:

VARIABLES	Total[1]	AZVUDINE N = 143[1]	PLACEBO N = 138[1]	p-value[2]
Viral load (D0)	9.971 ± 4.257 (11.553)	10.239 ± 3.985 (11.593)	9.694 ± 4.519 (11.394)	0.543
Viral load (D1)	8.631 ± 4.897 (10.756)	8.359 ± 5.083 (10.834)	8.909 ± 4.702 (10.717)	0.634
Viral load l(D3)	5.733 ± 5.503 (1.037)	4.452 ± 5.564 (969)	7.023 ± 5.146 (10.000)	**<0.001**
Viral load l(D5)	4.285 ± 5.189 (971)	3.016 ± 4.897 (0)	5.513 ± 5.187 (9.642)	**<0.001**
Viral load (D7)	1.773 ± 3.613 (63)	1.483 ± 3.516 (0)	1.987 ± 3.684 (967)	**<0.001**
Viral load D9)	1.276 ± 3.282 (0)	1.439 ± 3.653 (0)	1.214 ± 3.149 (29)	0.064
Viral load (D11)	556 ± 2.230 (0)	902 ± 2.637 (0)	462 ± 2.124 (0)	0.712
Viral load (D13)	334 ± 1.526 (0)	501 ± 536 (491)	294 ± 1.685 (0)	**0.002**
CTs(D0)	21.3 (18.9 - 24.8)	21.1 (19.0 - 24.4)	21.6 (18.5 - 25.2)	0.490
CTs(D1)	23.0 (20.0 - 26.4)	23.0 (20.0 - 26.6)	23.0 (20.0 - 25.9)	0.523
CTs(D3)	26.1 (23.1 - 29.8)	28.0 (23.0 - 31.0)	25.1 (23.1 - 27.8)	**<0.001**
CTs(D5)	27.9 (25.0 - 31.0)	31.0 (25.8 - 31.0)	26.0 (24.6 - 29.6)	**<0.001**
CTs(D7)	29.80 (26.83 - 31.00)	31.00 (27.65 - 31.00)	28.00 (26.80 - 31.00)	**<0.001**
CTs(D9)	31.00 (28.50 - 31.00)	31.00 (30.10 - 31.00)	29.80 (28.50 - 31.00)	0.062
CTs(D11)	31.00 (29.92 - 31.00)	31.00 (28.25 - 31.00)	31.00 (30.15 - 31.00)	0.708
CTs(D13)	31.00 (31.00 - 31.00)	29.20 (27.18 - 31.00)	31.00 (31.00 - 31.00)	0.002

[1]n (%); Median (IQR) / [2] Mann-Whitney test; Wilcoxon rank sum exact test

Supplementary Table 4: Time for improvement of symptoms.

VARIABLES	Total[1]	AZVUDINE N = 143[1]	PLACEBO N = 138[1]	p-value[2]
Myalgia (Qty.days)	2.07 ± 1.42 (1.00)	2.00 ± 1.30 (1.50)	2.13 ± 1.53 (1.00)	0.959
Lost olphate (Qty.days)	2.67 ± 1.84 (2.00)	2.17 ± 1.37 (2.00)	3.07 ± 2.09 (3.00)	0.107
Lost palate (Qty.days)	2.48 ± 1.78 (2.00)	1.96 ± 1.10 (2.00)	2.94 ± 2.12 (2.50)	0.050
Diarrhea (Qty.days)	2.08 ± 1.51 (1.50)	2.18 ± 1.59 (1.50)	1.98 ± 1.44 (1.50)	0.706
Dizziness (Qty.days)	1.97 ± 1.31 (1.00)	1.69 ± 1.17 (1.00)	2.23 ± 1.41 (2.00)	0.095
Fever (Qty.days)	1.26 ± 0.60 (1.00)	1.29 ± 0.64 (1.00)	1.24 ± 0.56 (1.00)	0.896
Chill (Qty.days)	1.78 ± 1.04 (1.00)	1.61 ± 0.86 (1.00)	2.00 ± 1.20 (2.00)	0.213
Sore throat (Qty.days)	2.08 ± 1.39 (2.00)	1.88 ± 1.18 (2.00)	2.26 ± 1.55 (2.00)	0.367
Coryza (Qty.days)	2.54 ± 1.97 (2.00)	2.24 ± 1.66 (2.00)	2.80 ± 2.19 (2.00)	0.207
Dyspnea (Qty.days)	2.52 ± 1.80 (2.00)	2.18 ± 1.17 (2.00)	2.89 ± 2.26 (2.00)	0.328
Tachypnea (Qty.days)	1.60 ± 0.85 (1.00)	1.50 ± 0.92 (1.00)	1.71 ± 0.77 (2.00)	0.251

Nausea (Qty.days)	1.89 ± 1.43 (1.00)	1.84 ± 1.14 (1.00)	1.94 ± 1.70 (1.00)	0.639
Vomit (Qty.days)	1.60 ± 1.44 (1.00)	1.40 ± 0.63 (1.00)	1.90 ± 2.18 (1.00)	0.973
Abdominal pain (Qty.days)	2.32 ± 2.09 (1.00)	2.22 ± 1.53 (2.00)	2.40 ± 2.45 (1.00)	0.413
Disability march (Qty.days)	2.00 ± 1.73 (1.00)	2.67 ± 2.89 (1.00)	1.67 ± 1.03 (1.00)	0.877

[1]n (%); Median (IQR) / [2]Mann-Whitney test Wilcoxon rank sum test

Supplementary Table 5: Comorbidities

VARIABLES	**N**	**Total[1]**	Azvudine N = 143[1]	Placebo N = 138[1]	**p-value[2]**
Insomnia	281	99 (35%)	55 (38%)	44 (32%)	0.249
Arterial hypertension	281	83 (30%)	44 (31%)	39 (28%)	0.645
Constipation	281	44 (16%)	18 (13%)	26 (19%)	0.149
Gastritis	281	38 (14%)	22 (15%)	16 (12%)	0.353
Labyrinthitis	281	35 (12%)	21 (15%)	14 (10%)	0.249
Obesity	281	31 (11%)	18 (13%)	13 (9.4%)	0.397
Hepatic steatosis	281	23 (8.2%)	14 (9.8%)	9 (6.5%)	0.318
Smoker	281	22 (7.8%)	12 (8.4%)	10 (7.2%)	0.721
Type 2 diabetes (non-insulin dependent)	281	20 (7.1%)	8 (5.6%)	12 (8.7%)	0.312
Irritable bowel syndrome	281	17 (6.0%)	12 (8.4%)	5 (3.6%)	0.094
Alcoholic	281	18 (6.4%)	8 (5.6%)	10 (7.2%)	0.572
Rheumatic disease	281	18 (6.4%)	9 (6.3%)	9 (6.5%)	0.938
Bronchitis	281	15 (5.3%)	10 (7.0%)	5 (3.6%)	0.209
Cardiac arrhythmia	281	14 (5.0%)	8 (5.6%)	6 (4.3%)	0.631
Kidney stone	281	14 (5.0%)	7 (4.9%)	7 (5.1%)	0.946
Osteoporosis	281	13 (4.6%)	4 (2.8%)	9 (6.5%)	0.137
Asthma	281	7 (2.5%)	4 (2.8%)	3 (2.2%)	>0.999
Type 1 diabetes (insulin dependent)	281	7 (2.5%)	4 (2.8%)	3 (2.2%)	>0.999
Cardiac arrhythmia	281	6 (2.1%)	5 (3.5%)	1 (0.7%)	0.214
Endometriosis	281	4 (1.4%)	4 (2.8%)	0 (0%)	0.123
Coronary angioplasty (stent)	281	4 (1.4%)	1 (0.7%)	3 (2.2%)	0.364
Angina	281	3 (1.1%)	1 (0.7%)	2 (1.4%)	0.617
Cardiac revascularization	281	2 (0.7%)	1 (0.7%)	1 (0.7%)	>0.999
Stroke	281	1 (0.4%)	1 (0.7%)	0 (0%)	>0.999

Supplementary Table 6: Total values of urea, creatinine and uric acid.

VARIÁVEIS	**N**	**Total[1]**	AZVUDINE<br>N = 143[1]	PLACEBO<br>N = 138[1]	**p-value[2]**
Urea (D1)	270	28 ± 9 (28)	27 ± 9 (27)	28 ± 10 (29)	0.356
Urea (D3)	218	31 ± 10 (29)	31 ± 9 (31)	31 ± 11 (29)	0.437
Urea (D5)	203	31 ± 11 (28)	30 ± 10 (28)	31 ± 12 (30)	0.800
Urea (D7)	149	31 ± 11 (30)	31 ± 10 (30)	31 ± 12 (30)	0.888
Urea (D9)	94	31 ± 11 (29)	30 ± 9 (29)	32 ± 11 (30)	0.494
Urea (D11)	56	31 ± 10 (30)	30 ± 11 (28)	31 ± 10 (30)	0.570
Urea (D13)	33	30 ± 9 (31)	30 ± 9 (31)	30 ± 10 (31)	0.981
Urea (D15)	251	31 ± 10 (29)	31 ± 10 (30)	30 ± 11 (29)	0.402
Urea (D28)	275	28 ± 9 (27)	28 ± 8 (27)	28 ± 10 (27)	0.594
Creatinine (D0)	281	0.84 (0.72 - 1.05)	0.84 (0.75 - 1.06)	0.85 (0.70 - 1.05)	0.712
Creatinine (D1)	281	0.89 ± 0.25 (0.84)	0.89 ± 0.24 (0.84)	0.88 ± 0.26 (0.85)	0.712
Creatinine (D3)	270	0.92 ± 0.60 (0.87)	0.89 ± 0.25 (0.85)	0.96 ± 0.82 (0.87)	0.747
Creatinine (D5)	219	0.82 ± 0.19 (0.82)	0.83 ± 0.22 (0.82)	0.81 ± 0.17 (0.81)	0.754
Creatinine (D7)	203	0.85 ± 0.23 (0.83)	0.84 ± 0.24 (0.81)	0.86 ± 0.23 (0.84)	0.493
Creatinine (D9)	149	1.07 ± 2.72 (0.84)	1.50 ± 4.60 (0.84)	0.84 ± 0.20 (0.85)	0.911
Creatinine (D11)	94	0.85 ± 0.24 (0.82)	0.83 ± 0.24 (0.78)	0.86 ± 0.24 (0.83)	0.584
Creatinine (D13)	56	0.81 ± 0.25 (0.81)	0.85 ± 0.18 (0.84)	0.81 ± 0.26 (0.80)	0.489
Creatinine (D15)	33	0.84 ± 0.27 (0.85)	0.79 ± 0.25 (0.89)	0.85 ± 0.28 (0.82)	>0.999
Creatinine (D28)	252	0.85 ± 0.21 (0.84)	0.86 ± 0.21 (0.84)	0.84 ± 0.20 (0.84)	0.917
Uric acid (D1)	270	4.02 ± 1.49 (3.90)	4.12 ± 1.35 (4.00)	3.92 ± 1.62 (3.79)	0.183
Uric acid (D3)	218	3.90 ± 1.31 (3.80)	4.01 ± 1.29 (3.90)	3.80 ± 1.33 (3.80)	0.283
Uric acid (D5)	203	4.00 ± 1.41 (3.97)	4.07 ± 1.36 (4.00)	3.94 ± 1.44 (3.90)	0.590
Uric acid (D7)	149	4.15 ± 1.41 (4.10)	4.20 ± 1.34 (4.05)	4.13 ± 1.45 (4.10)	0.858
Uric acid (D9)	94	4.45 ± 1.57 (4.41)	4.38 ± 1.50 (4.20)	4.48 ± 1.60 (4.50)	0.555
Uric acid D11)	56	4.71 ± 1.67 (4.44)	4.16 ± 0.88 (3.90)	4.84 ± 1.79 (4.60)	0.132
Uric acid (D13)	33	4.76 ± 1.83 (4.70)	3.95 ± 0.64 (3.95)	4.94 ± 1.96 (5.00)	0.168
Uric acid (D15)	251	4.47 ± 1.49 (4.43)	4.62 ± 1.41 (4.48)	4.31 ± 1.57 (4.40)	0.153
Uric acid (D28)	275	4.73 ± 1.50 (4.70)	4.88 ± 1.40 (5.00)	4.57 ± 1.59 (4.60)	0.032
Uric acid (D1)	270	4.02 ± 1.49 (3.90)	4.12 ± 1.35 (4.00)	3.92 ± 1.62 (3.79)	0.183

VARIÁVEIS	**N**	**Total[1]**	AZVUDINE<br><br>N = 143[1]	PLACEBO<br><br>N = 138[1]	**p-value[2]**
Uric acid (D3)	218	3.90 ± 1.31 (3.80)	4.01 ± 1.29 (3.90)	3.80 ± 1.33 (3.80)	0.283
Uric acid (D5)	203	4.00 ± 1.41 (3.97)	4.07 ± 1.36 (4.00)	3.94 ± 1.44 (3.90)	0.590
Uric acid (D7)	149	4.15 ± 1.41 (4.10)	4.20 ± 1.34 (4.05)	4.13 ± 1.45 (4.10)	0.858
Uric acid (D9)	94	4.45 ± 1.57 (4.41)	4.38 ± 1.50 (4.20)	4.48 ± 1.60 (4.50)	0.555

[1]Median (IQR); [2] Mann-Whitney test

Supplementary Table 7: values of AST/TGO, ALT/TGP, GGT and BT.

VARIABLES	N	**Total[1]**	AZVUDINE N = 143[1]	PLACEBO N = 138[1]	**p-value[2]**
AST/TGO(D0)	281	34 ± 21 (31)	34 ± 14 (32)	34 ± 26 (29)	**0.015**
AST/TGO(D1)	271	33 ± 15 (31)	33 ± 13 (32)	32 ± 17 (30)	0.232
AST/TGO (D3)	219	34 ± 20 (30)	34 ± 20 (31)	33 ± 20 (30)	0.345
AST/TGO (D5)	204	34 ± 20 (30)	35 ± 22 (31)	32 ± 17 (28)	0.057
AST/TGO (D7)	149	31 ± 10 (29)	34 ± 9 (32)	29 ± 10 (28)	**<0.001**
AST/TGO (D9)	95	34 ± 30 (28)	37 ± 26 (32)	33 ± 31 (27)	0.039
AST/TGO (D11)	56	31 ± 13 (28)	34 ± 10 (31)	31 ± 14 (27)	0.131
AST/TGO (D13)	33	30 ± 15 (28)	40 ± 20 (34)	28 ± 14 (28)	0.141
AST/TGO (D15)	252	32 ± 16 (29)	31 ± 15 (29)	32 ± 18 (28)	0.846
AST/TGO (D28)	275	31 ± 16 (29)	31 ± 14 (29)	30 ± 17 (28)	0.062
ALT/TGP(D0)	281	27 ± 20 (22)	28 ± 18 (23)	26 ± 23 (21)	0.052
ALT/TGP(D1)	271	27 ± 17 (23)	27 ± 17 (23)	26 ± 18 (22)	0.567
ALT/TGP (D3)	219	31 ± 21 (24)	30 ± 19 (23)	32 ± 23 (24)	0.693
ALT/TGP (D5)	204	33 ± 30 (24)	34 ± 33 (26)	32 ± 26 (23)	0.218
ALT/TGP (D7)	149	32 ± 21 (26)	34 ± 25 (28)	30 ± 19 (24)	0.320
ALT/TGP (D9)	95	36 ± 60 (24)	32 ± 20 (27)	38 ± 69 (24)	0.434
ALT/TGP (D11)	56	31 ± 37 (21)	29 ± 20 (22)	32 ± 40 (21)	0.726
ALT/TGP (D13)	33	27 ± 18 (22)	35 ± 36 (22)	25 ± 13 (23)	0.944
ALT/TGP (D15)	252	29 ± 19 (24)	30 ± 21 (24)	28 ± 17 (23)	0.344
ALT/TGP (D28)	275	26 ± 19 (21)	27 ± 19 (21)	25 ± 18 (21)	0.508
GGT(D1)	271	43 ± 37 (29)	47 ± 41 (32)	38 ± 33 (28)	**0.027**
GGT(D3)	219	47 ± 43 (32)	51 ± 46 (37)	43 ± 39 (29)	0.059
GGT(D5)	204	46 ± 39 (34)	50 ± 42 (37)	42 ± 35 (30)	0.054
GGT(D7)	149	43 ± 34 (32)	50 ± 39 (40)	39 ± 30 (30)	0.059
GGT(D9)	95	48 ± 61 (32)	51 ± 40 (42)	46 ± 68 (30)	0.285
GGT(D11)	65	41 ± 58 (28)	47 ± 51 (32)	39 ± 60 (25)	0.384
GGT(D13)	33	42 ± 39 (31)	67 ± 70 (32)	37 ± 28 (31)	0.315
GGT(D15)	252	41 ± 31 (32)	44 ± 32 (34)	37 ± 29 (31)	**0.029**
GGT(D28)	275	39 ± 32 (29)	43 ± 35 (32)	35 ± 28 (26)	**0.011**

VARIABLES	N	Total[1]	AZVUDINE N = 143[1]	PLACEBO N = 138[1]	p-value[2]
BT(D0)	281	0.59 ± 0.28 (0.54)	0.60 ± 0.31 (0.55)	0.58 ± 0.25 (0.54)	0.716
BT(D1)	270	0.63 ± 0.30 (0.57)	0.63 ± 0.30 (0.59)	0.64 ± 0.31 (0.55)	0.985
BT(D3)	219	0.61 ± 0.28 (0.56)	0.62 ± 0.27 (0.58)	0.60 ± 0.30 (0.55)	0.292
BT(D5)	203	0.65 ± 0.30 (0.61)	0.71 ± 0.32 (0.66)	0.60 ± 0.26 (0.57)	**0.005**
BT(D7)	149	0.65 ± 0.25 (0.61)	0.66 ± 0.23 (0.63)	0.64 ± 0.26 (0.59)	0.530
BT(D9)	95	0.64 ± 0.24 (0.61)	0.68 ± 0.25 (0.65)	0.63 ± 0.23 (0.61)	0.407
BT(D11)	56	0.67 ± 0.21 (0.65)	0.66 ± 0.22 (0.57)	0.68 ± 0.21 (0.67)	0.536
BT(D15)	33	0.63 ± 0.25 (0.58)	0.62 ± 0.17 (0.56)	0.63 ± 0.27 (0.58)	0.981
BT(D28)	251	0.96 ± 4.64 (0.63)	0.67 ± 0.31 (0.62)	1.27 ± 6.62 (0.63)	0.834

[1]Median (IQR); [2] Mann-Whitney test

Supplementary Table 8: Biochemical analysis of blood.

VARIABLES	N	Total[1]	AZVUDINE N = 143[1]	PLACEBO N = 138[1]	p-value[2]
HGT(D0)	281	99 (88 - 105)	100 (92 - 108)	98 (87 - 103)	**0.014**
HGT(D1)	281	106 (93 - 123)	110 (96 - 124)	102 (93 - 121)	0.27
HGT(D2)	281	106 (95 - 131)	107 (95 - 132)	106 (95 - 131)	0.961
HGT(D3)	281	106 (97 - 131)	105 (98 - 130)	108 (97 - 134)	0.983
HGT(D4)	281	105 (97 - 132)	105 (97 - 130)	106 (97 - 133)	0.917
HGT(D5)	281	106 (95 - 130)	103 (95 - 126)	108 (96 - 131)	0.574
HGT(D6)	281	107 (97 - 126)	106 (97 - 126)	107 (96 - 126)	0.973
HGT(D7)	281	106 (98 - 137)	102 (97 - 134)	108 (98 - 138)	0.526
HGT(D8)	281	107 (97 - 132)	103 (93 - 132)	111 (98 - 129)	0.307
HGT(D9)	281	108 (97 - 136)	106 (98 - 136)	110 (97 - 135)	0.706
HGT(D10)	281	103 (95 - 120)	101 (94 - 143)	104 (95 - 118)	0.917
HGT(D11)	281	103 (97 - 129)	104 (101 - 128)	102 (94 - 128)	0.209
HGT(D12)	281	119 (101 - 138)	124 (116 - 164)	114 (100 - 135)	0.139

HGT(D13)	281	126 (112 - 150)	144 (130 - 188)	120 (106 - 142)	0.029
HGT(D14)	281	117 (102 - 136)	124 (102 - 138)	117 (100 - 136)	0.954
Hemoglobin (D0)	280	13.52 ± 1.38 (13.40)	13.64 ± 1.41 (13.45)	13.39 ± 1.34 (13.30)	0.134
Hemoglobin (D1)	273	13.48 ± 1.75 (13.30)	13.60 ± 2.04 (13.30)	13.36 ± 1.39 (13.30)	0.414
Hemoglobin (D3)	218	13.64 ± 2.15 (13.40)	13.60 ± 1.49 (13.55)	13.67 ± 2.62 (13.40)	0.295
Hemoglobin (D5)	206	13.44 ± 1.37 (13.40)	13.70 ± 1.39 (13.70)	13.22 ± 1.32 (13.10)	**0.016**
Hemoglobin (D7)	149	13.44 ± 1.39 (13.30)	13.64 ± 1.45 (13.75)	13.34 ± 1.36 (13.00)	0.223
Hemoglobin (D9)	90	13.37 ± 1.36 (13.10)	13.79 ± 1.36 (13.55)	13.24 ± 1.34 (13.00)	0.116
Hemoglobin (D11)	56	13.35 ± 2.75 (13.20)	14.75 ± 5.50 (13.20)	13.01 ± 1.39 (13.20)	0.620
Hemoglobin (D13)	33	12.64 ± 1.50 (12.60)	12.85 ± 0.70 (12.75)	12.59 ± 1.64 (12.30)	0.674
Hemoglobin (D15)	251	13.20 ± 1.92 (13.20)	13.37 ± 1.69 (13.50)	13.02 ± 2.13 (13.00)	0.141
Hemoglobin (D28	275	13.01 ± 1.78 (13.00)	13.15 ± 1.85 (13.10)	12.86 ± 1.69 (12.80)	0.094
Hematocrite (D0)	280	40.8 ± 3.9 (40.8)	41.2 ± 4.0 (41.1)	40.4 ± 3.8 (40.4)	0.081
Hematocrite (D1)	273	40.4 ± 4.0 (40.3)	40.5 ± 4.0 (40.3)	40.4 ± 4.0 (40.3)	0.788
Hematocrite (D3)	218	40.7 ± 4.5 (40.8)	40.8 ± 5.5 (41.2)	40.6 ± 3.4 (40.2)	0.171
Hematocrite (D5)	206	40.6 ± 3.9 (40.5)	41.5 ± 3.9 (41.3)	40.0 ± 3.8 (39.7)	0.006
Hematocrite (D7)	149	40.5 ± 4.0 (39.7)	41.0 ± 3.9 (40.9)	40.3 ± 4.0 (39.3)	0.224
Hematocrite (D9)	90	40.4 ± 3.9 (39.7)	41.3 ± 3.5 (40.9)	40.1 ± 4.0 (39.4)	0.195
Hematocrite (D11)	56	39.6 ± 3.8 (39.7)	39.6 ± 3.1 (39.4)	39.6 ± 4.0 (40.1)	0.820
Hematocrite (D13)	33	38.6 ± 4.3 (38.0)	38.0 ± 2.3 (37.7)	38.7 ± 4.7 (38.0)	0.640
Hematocrite (D15)	251	40.1 ± 4.6 (40.0)	40.6 ± 3.8 (40.6)	39.6 ± 5.3 (39.6)	0.117
Hematocrite (D28)	275	39.3 ± 5.2 (39.4)	39.8 ± 5.3 (40.1)	38.9 ± 4.9 (39.0)	0.068
Total Protein (D1)	261	6.73 ± 1.90 (7.20)	6.70 ± 1.88 (7.20)	6.75 ± 1.93 (7.20)	0.420
Total Protein (D15)	234	9.68 ± 45.81 (7.00)	6.72 ± 1.65 (7.00)	12.68 ± 65.05 (7.00)	0.500
Total Protein (D28)	272	6.87 ± 1.30 (7.00)	6.93 ± 1.13 (7.00)	6.82 ± 1.45 (7.00)	0.585
Globulin (D1)	261	2.76 ± 0.86 (2.90)	2.75 ± 0.85 (2.90)	2.77 ± 0.87 (2.90)	0.840
Globulin (D15)	234	3.88 ± 17.82 (2.80)	2.76 ± 0.76 (2.80)	5.02 ± 25.30 (2.80)	0.297
Globulin (D28)	272	2.75 ± 0.61 (2.80)	2.79 ± 0.56 (2.80)	2.71 ± 0.65 (2.80)	0.555
Albumin (D1)	261	3.94 ± 1.14 (4.20)	3.91 ± 1.14 (4.20)	3.98 ± 1.14 (4.20)	0.234
Albumin (D15)	234	3.97 ± 0.98 (4.10)	3.96 ± 0.99 (4.12)	3.98 ± 0.99 (4.10)	0.753
Albumin (D28)	272	4.14 ± 0.76 (4.20)	4.17 ± 0.62 (4.20)	4.11 ± 0.89 (4.20)	0.872

[1]Median (IQR); [2]Mann-Whitney test

Despite having significant data, the values are within the normal range. Total protein and globulin increased in D15 justifying an increase in antibody production in infectious diseases. However, no changes was observed for uric acid.

Supplementary Table 9: Analysis of blood electrolytes.

VARIABLES	N	Total[1]	AZVUDINE N = 143[1]	PLACEBO N = 138[1]	p-value[2]
Sodium (D1)	270	136.78 ± 14.76 (138.00)	138.23 ± 2.73 (138.00)	135.29 ± 20.78 (138.00)	0.585
Sodium (D3)	217	135.60 ± 18.83 (138.00)	133.78 ± 23.34 (138.00)	137.27 ± 13.31 (138.67)	0.051
Sodium (D5)	203	135.5 ± 18.3 (138.2)	134.6 ± 20.5 (138.0)	136.3 ± 16.3 (138.9)	0.043
Sodium (D7)	149	136.2 ± 16.2 (138.0)	137.4 ± 2.9 (138.0)	135.5 ± 20.0 (138.0)	0.264
Sodium (D9)	93	135.5 ± 14.5 (137.0)	136.4 ± 3.6 (136.7)	135.2 ± 16.9 (137.1)	0.373
Sodium (D11)	56	137.16 ± 3.32 (137.52)	137.03 ± 3.99 (138.43)	137.19 ± 3.19 (137.00)	0.509
Sodium (D13)	33	129 ± 33 (138)	138 ± 3 (138)	127 ± 37 (137)	0.451
Sodium (D15)	251	135.5 ± 17.5 (138.0)	136.3 ± 12.5 (137.7)	134.6 ± 21.6 (138.0)	0.165
Sodium (D28)	274	133.2 ± 24.5 (138.0)	132.8 ± 25.7 (138.0)	133.7 ± 23.1 (138.0)	0.976
Potassium (D1)	267	4.05 ± 1.48 (4.30)	4.02 ± 1.61 (4.30)	4.09 ± 1.33 (4.28)	0.752
Potassium (D3)	217	3.95 ± 1.53 (4.27)	3.83 ± 1.63 (4.24)	4.05 ± 1.42 (4.29)	0.556
Potassium (D5)	203	4.04 ± 1.26 (4.25)	4.13 ± 1.09 (4.40)	3.96 ± 1.39 (4.12)	0.027
Potassium (D7)	149	4.13 ± 1.08 (4.30)	4.33 ± 0.77 (4.50)	4.02 ± 1.21 (4.29)	0.085
Potassium (D9)	93	4.42 ± 1.67 (4.50)	5.00 ± 1.90 (4.50)	4.20 ± 1.54 (4.46)	0.386
Potassium (D11)	56	4.27 ± 1.35 (4.47)	4.03 ± 1.40 (4.40)	4.33 ± 1.35 (4.60)	0.433

Potassium (D13)	33	4.26 ± 1.65 (4.60)	4.38 ± 0.47 (4.37)	4.23 ± 1.82 (4.63)	0.575
Potassium (D15)	251	4.03 ± 1.67 (4.35)	4.00 ± 1.88 (4.32)	4.06 ± 1.44 (4.39)	0.386
Potassium (D28)	272	4.11 ± 1.65 (4.30)	4.02 ± 1.78 (4.30)	4.22 ± 1.51 (4.30)	0.854
Chlorine (D1)	265	105.7 ± 73.1 (102.0)	107.2 ± 71.5 (102.0)	104.3 ± 75.0 (102.0)	0.292
Chlorine (D3)	217	99.6 ± 10.6 (101.5)	98.7 ± 14.5 (101.8)	100.4 ± 4.8 (101.0)	0.811
Chlorine (D5)	203	99.9 ± 4.1 (100.4)	99.5 ± 4.1 (100.0)	100.2 ± 4.1 (101.5)	0.122
Chlorine (D7)	149	99.7 ± 4.4 (100.0)	99.5 ± 4.5 (100.0)	99.9 ± 4.5 (100.0)	0.658
Chlorine (D9)	93	100.4 ± 4.2 (101.0)	101.0 ± 4.1 (102.0)	100.2 ± 4.3 (101.0)	0.402
Chlorine (D11)	56	100.6 ± 4.2 (101.4)	101.5 ± 3.9 (103.7)	100.4 ± 4.3 (101.1)	0.368
Chlorine (D13)	33	101.6 ± 3.6 (102.0)	100.9 ± 3.0 (101.0)	101.8 ± 3.7 (102.0)	0.574
Chlorine (D15)	251	99.9 ± 7.6 (101.0)	100.4 ± 4.2 (101.4)	99.4 ± 10.1 (101.0)	0.830
Chlorine (D28)	274	100.8 ± 9.6 (102.0)	100.7 ± 9.4 (102.0)	100.8 ± 9.9 (102.0)	0.985
Calcium (D1)	268	9.20 ± 1.18 (9.25)	9.31 ± 0.75 (9.30)	9.09 ± 1.49 (9.21)	0.500
Calcium (D3)	217	9.73 ± 5.91 (9.33)	9.41 ± 0.48 (9.40)	10.02 ± 8.19 (9.21)	**0.049**
Calcium (D5)	203	9.21 ± 0.85 (9.24)	9.29 ± 0.58 (9.30)	9.14 ± 1.01 (9.20)	0.135
Calcium (D7)	149	9.28 ± 0.51 (9.30)	9.35 ± 0.57 (9.35)	9.24 ± 0.48 (9.28)	0.268
Calcium (D9)	93	9.26 ± 0.52 (9.20)	9.38 ± 0.65 (9.30)	9.21 ± 0.46 (9.20)	0.186
Calcium (D11)	56	9.33 ± 0.52 (9.30)	9.20 ± 0.32 (9.14)	9.37 ± 0.56 (9.30)	0.438
Calcium (D13)	33	9.29 ± 0.60 (9.30)	9.03 ± 0.48 (9.05)	9.35 ± 0.62 (9.30)	0.242
Calcium (D15)	251	9.18 ± 0.98 (9.20)	9.31 ± 0.55 (9.27)	9.04 ± 1.27 (9.17)	**0.042**
Calcium (D28)	273	9.27 ± 0.94 (9.30)	9.34 ± 0.94 (9.40)	9.19 ± 0.94 (9.20)	**0.021**

[1]*Median (IQR);* [2]*Mann-Whitney test*

The electrolyte values fluctuate, but within the normal range, demonstrating good safety.

Supplementary Table 10: Demonstration of inflammatory marker values during the study days.

VARIABLES	N	**Total[1]**	AZVUDINE N = 143[1]	PLACEBO N = 138[1]	**p-value[2]**
US PCR(D1)	263	4.6 ± 8.1 (2.0)	3.6 ± 5.3 (1.6)	5.6 ± 10.2 (3.3)	0.161
US PCR (D15)	238	3.2 ± 4.6 (0.9)	2.6 ± 3.6 (0.5)	3.7 ± 5.3 (1.4)	0.024
US PCR (D28)	274	2.51 ± 3.14 (0.68)	2.43 ± 3.16 (0.75)	2.60 ± 3.13 (0.55)	0.996
D Dimero(D1)	281	300 ± 298 (270)	289 ± 285 (270)	311 ± 312 (270)	0.696
CPK-MB(D1)	270	7 ± 9 (2)	7 ± 10 (1)	7 ± 9 (2)	0.815
CPK-MB(D15)	239	6 ± 9 (0)	5 ± 8 (0)	7 ± 10 (0)	0.451
CPK-MB(D28)	274	5 ± 11 (0)	5 ± 10 (0)	5 ± 12 (0)	0.868
Troponin I(D1)	257	1.67 ± 3.80 (0.40)	1.69 ± 3.52 (0.40)	1.65 ± 4.07 (0.35)	0.501
Troponin I (D15)	228	1.63 ± 3.17 (0.65)	1.58 ± 2.67 (0.90)	1.67 ± 3.63 (0.50)	0.370
Troponin I (D28)	271	1.94 ± 3.35 (1.10)	2.15 ± 3.83 (1.20)	1.71 ± 2.74 (0.90)	0.159
TTPA (D1)	273	29 ± 10 (33)	29 ± 10 (33)	29 ± 10 (33)	0.859
TTPA (D15)	252	28 ± 10 (31)	27 ± 11 (32)	29 ± 9 (31)	0.919
TTPA (D28)	275	32.2 ± 16.7 (33.0)	31.7 ± 6.6 (33.0)	32.7 ± 22.9 (33.0)	0.467
TAP (D1)	273	18.2 ± 100.2 (13.0)	12.2 ± 3.5 (13.0)	24.4 ± 142.5 (13.0)	0.782
TAP (D15)	252	16.4 ± 71.1 (13.0)	20.5 ± 99.4 (13.0)	12.2 ± 3.6 (13.0)	0.599
TAP (D28)	275	13.03 ± 2.24 (13.00)	13.20 ± 1.71 (13.00)	12.86 ± 2.68 (13.00)	0.032
ESR (D1)	260	20 ± 19 (11)	20 ± 20 (10)	20 ± 17 (13)	0.357
ESR (D15)	244	17 ± 19 (6)	15 ± 17 (5)	19 ± 20 (7)	0.168
ESR (D28)	273	19 ± 18 (9)	19 ± 19 (9)	19 ± 18 (11)	0.784
Platelets (D0)	280	228,165 ± 63,844 (225,000)	232,710 ± 66,141 (234,500)	223,488 ± 61,279 (215,500)	0.190
Platelets (D1)	273	238,097 ± 65,987 (230,000)	244,578 ± 67,834 (241,000)	231,471 ± 63,612 (219,000)	0.055
Platelets (D3)	218	257,088 ± 71,750 (251,500)	265,273 ± 80,414 (263,500)	249,621 ± 62,241 (242,000)	0.099
Platelets (D5)	206	264,124 ± 70,517 (264,350)	272,075 ± 76,534 (269,500)	257,707 ± 64,893 (258,000)	0.142
Platelets (D7)	149	272,596 ± 74,070 (268,000)	285,212 ± 81,139 (281,500)	265,833 ± 69,485 (262,000)	0.144
Platelets (D9)	90	268,528 ± 72,885 (269,500)	246,750 ± 84,726 (249,250)	275,574 ± 67,827 (273,000)	0.112
Platelets (D11)	56	270,971 ± 70,057 (258,500)	265,273 ± 78,216 (253,000)	272,364 ± 68,808 (278,000)	0.657
Platelets (D13)	33	276,394 ± 107,343 (266,000)	255,167 ± 74,516 (265,500)	281,111 ± 113,959 (266,000)	0.762
Platelets (D15)	251	266,719 ± 71,390 (263,000)	277,832 ± 74,732 (275,000)	254,968 ± 65,964 (251,000)	**0.016**
Platelets (D28)	275	238,632 ± 61,406 (235,000)	243,174 ± 63,990 (240,000)	233,853 ± 58,421 (230,000)	0.195

[1]Median (IQR); [2]Mann-Whitney test

Supplementary Table 11: Demonstration of white series values and immunological markers during the study days

VARIABLES	N	**Total[1]**	AZVUDINE N = 143[1]	PLACEBO N = 138[1]	**p-value[2]**
Leukocytes (D0)	280	6,574 ± 4,055 (6,100)	6,361 ± 2,380 (5,900)	6,792 ± 5,249 (6,400)	0.672
Leukocytes (D1)	273	6,837 ± 5,904 (6,000)	6,441 ± 2,247 (6,100)	7,243 ± 8,079 (6,000)	0.637
Leukocytes (D3)	218	8,224 ± 3,213 (8,100)	8,541 ± 3,221 (8,250)	7,935 ± 3,193 (8,000)	0.177
Leukocytes (D5)	206	8,536 ± 5,871 (7,950)	8,174 ± 2,926 (8,400)	8,829 ± 7,447 (7,550)	0.971
Leukocytes (D7)	149	8,462 ± 4,344 (8,200)	7,905 ± 2,877 (7,950)	8,761 ± 4,944 (8,300)	0.302
Leukocytes (D9)	90	8,207 ± 2,968 (7,800)	7,161 ± 2,895 (7,200)	8,546 ± 2,932 (7,950)	0.089
Leukocytes (D11)	56	8,681 ± 7,709 (7,550)	6,555 ± 2,476 (6,400)	9,201 ± 8,455 (7,600)	0.051
Leukocytes (D13)	33	7,064 ± 1,655 (7,200)	6,350 ± 1,540 (6,550)	7,222 ± 1,665 (7,600)	0.199
Leukocytes (D15)	251	8,062 ± 5,663 (7,600)	8,092 ± 2,901 (7,900)	8,030 ± 7,573 (7,400)	0.130

Leukocytes (D28)	275	6,702 ± 5,942 (6,000)	7,275 ± 8,096 (6,200)	6,099 ± 1,742 (5,800)	0.280
Neutrophils (D0)cells/mm3	280	3,843 ± 1,881 (3,460)	3,775 ± 1,925 (3,368)	3,914 ± 1,839 (3,624)	0.469
Neutrophils (D1)cells/mm3	273	3,750 ± 1,976 (3,245)	3,753 ± 1,808 (3,504)	3,747 ± 2,141 (3,082)	0.476
Neutrophils (D3)cells/mm3	218	5,651 ± 2,930 (5,621)	5,804 ± 2,962 (5,818)	5,510 ± 2,907 (5,504)	0.428
Neutrophils (D5)cells/mm3	206	4,885 ± 2,422 (4,561)	4,904 ± 2,395 (4,916)	4,871 ± 2,454 (4,294)	0.833
Neutrophils (D7)cells/mm3	149	5,151 ± 2,210 (4,708)	4,867 ± 2,110 (4,214)	5,303 ± 2,258 (4,897)	0.315
Neutrophils (D9)cells/mm3	90	4,837 ± 2,633 (4,359)	4,196 ± 2,526 (4,054)	5,044 ± 2,651 (4,430)	0.124
Neutrophils (D11)cells/mm3	56	4,567 ± 2,354 (4,580)	3,842 ± 2,254 (3,219)	4,744 ± 2,368 (4,585)	0.232
Neutrophils (D13)cells/mm3	33	4,176 ± 1,475 (4,339)	4,037 ± 1,660 (4,206)	4,207 ± 1,464 (4,339)	0.838
Neutrophils (D15)cells/mm3	251	4,652 ± 2,145 (4,293)	4,861 ± 2,317 (4,361)	4,431 ± 1,931 (4,256)	0.293
Neutrophils (D28)cells/mm3	275	3,525 ± 1,575 (3,300)	3,648 ± 1,759 (3,342)	3,395 ± 1,350 (3,226)	0.391
Neutrophils (D0)%	280	58 ± 12 (59)	57 ± 12 (59)	59 ± 12 (59)	0.196
Neutrophils (D1)%	273	56 ± 12 (57)	56 ± 11 (57)	56 ± 13 (56)	0.870
Neutrophils (D3)%	218	65 ± 15 (68)	65 ± 15 (68)	65 ± 16 (67)	0.956
Neutrophils (D5)%	206	58 ± 13 (59)	57 ± 13 (57)	58 ± 13 (59)	0.452
Neutrophils (D7)%	149	60 ± 12 (60)	59 ± 12 (58)	61 ± 11 (60)	0.186
Neutrophils (D9)%	90	56 ± 13 (56)	55 ± 15 (55)	57 ± 12 (56)	0.725
Neutrophils (D11)%	56	57 ± 12 (58)	54 ± 16 (53)	58 ± 11 (58)	0.688
Neutrophils (D13)%	33	58 ± 14 (60)	61 ± 18 (63)	57 ± 13 (59)	0.560
Neutrophils (D15)%	251	58 ± 12 (59)	58 ± 12 (59)	58 ± 12 (59)	0.872
Neutrophils (D28)%	275	54 ± 12 (55)	55 ± 12 (55)	54 ± 12 (55)	0.507
Lymphocytes %(D0)	280	1,827 ± 692 (1,725)	1,861 ± 745 (1,732)	1,792 ± 634 (1,706)	0.400
Lymphocytes %(D1)	273	2,026 ± 706 (1,974)	2,042 ± 705 (2,012)	2,010 ± 709 (1,924)	0.497
Lymphocytes %(D3)	218	2,062 ± 895 (1,902)	2,116 ± 875 (2,044)	2,012 ± 914 (1,842)	0.196
Lymphocytes %(D5)	206	2,583 ± 970 (2,377)	2,600 ± 1,015 (2,458)	2,569 ± 937 (2,348)	0.870
Lymphocytes %(D7)	149	2,493 ± 906 (2,331)	2,595 ± 1,048 (2,400)	2,438 ± 821 (2,287)	0.495
Lymphocytes %(D9)	90	2,812 ± 2,425 (2,486)	3,204 ± 4,693 (2,316)	2,686 ± 918 (2,559)	0.132
Lymphocytes %(D11)	56	2,388 ± 860 (2,236)	2,338 ± 1,371 (1,931)	2,401 ± 704 (2,340)	0.119
Lymphocytes %(D13)	33	2,100 ± 687 (2,117)	1,740 ± 741 (1,686)	2,180 ± 662 (2,323)	0.189
Lymphocytes %(D15)	251	2,451 ± 1,081 (2,304)	2,528 ± 1,020 (2,394)	2,370 ± 1,140 (2,253)	0.183
Lymphocytes %(D28)	275	2,045 ± 626 (2,001)	2,046 ± 622 (2,005)	2,044 ± 632 (1,999)	0.997

[1]Median (IQR); [2]Mann-Whitney test

Supplementary Table 12: Objectives and outcomes

**OBJECTIVE AND OUTCOMES**	**p-value**
Score Reduction	>0,999
Cure time/absence of viral RNA	**<0.001**
Viral load negative time	**<0.001**
Days 1st Negative Conversion	**<0.001**
Days 2st Negative Conversion	**<0.001**
Medication use time	**<0.001**
Moment of viral load reduction	D3, D5, D9, D11
Participants who did not become viral load negative within 14 days	1,77%
